# Invasive apocrine carcinoma of the breast—a surgeon’s perspective on diagnosis, pitfalls, and evolving management options

**DOI:** 10.1093/jscr/rjaf921

**Published:** 2025-11-14

**Authors:** Rajshekhar C Jaka, Aishwarya Madasamy Swaminathan, Sunil Kumar Shetty

**Affiliations:** Department of Surgical Oncology, Manipal Hospitals, ITPL Main Road, Whitefield, Karnataka, Bangalore, 560066, India; Department of Surgical Oncology, Manipal Hospitals, ITPL Main Road, Whitefield, Karnataka, Bangalore, 560066, India; Department of General Surgery, Kasturba Medical College Mangalore, Manipal Academy of Higher Education, Karnataka, Manipal, 576104, India

**Keywords:** apocrine carcinoma, breast carcinoma, androgen receptor, triple negative, sentinel lymph node, oncoplastic surgery

## Abstract

Invasive apocrine carcinoma (IAC) is a rare histologic subtype of breast carcinoma that commonly shows an ER/PR/HER2 triple negative phenotype with strong androgen receptor (AR) expression. We report a case of a 58 year old woman with an incidentally detected left breast lesion, treated with breast conserving surgery and sentinel lymph node biopsy. Final pathology revealed a 1.7 × 1 × 1 cm invasive carcinoma with apocrine morphology, clear margins and five negative sentinel nodes; IHC showed ER/PR/HER2 negativity, AR strongly positive (90%), and low proliferation (Ki-67 8%). The patient received whole breast radiotherapy followed by systemic chemotherapy with paclitaxel and carboplatin. This report emphasizes diagnostic pitfalls (including positron emission tomography limitations and cytology traps), practical intraoperative decision-making, axillary management tailored to indolent biology, and the emerging role of AR directed and genomic guided therapies from a surgeon’s viewpoint. Awareness of these issues helps avoid overtreatment while preserving options for targeted systemic approaches.

## Introduction

Invasive apocrine carcinoma (IAC) is an uncommon histologic subtype of breast cancer with characteristic morphology and a distinctive immunophenotype; it has been variably estimated to represent under 1% of invasive breast cancers [1, 2]. Molecular profiling has identified a “molecular apocrine” subset that is typically ER/PR negative and frequently AR positive, creating therapeutic opportunities distinct from basal like triple negative disease [1, 3]. Although the natural history of IAC may be less aggressive than some basal triple negative breast cancers (TNBCs), often with lower proliferation indices, clinical management remains guided by multidisciplinary assessment and growing interest in AR targeted approaches [3, 4].

## Case presentation

**Figure 1:**
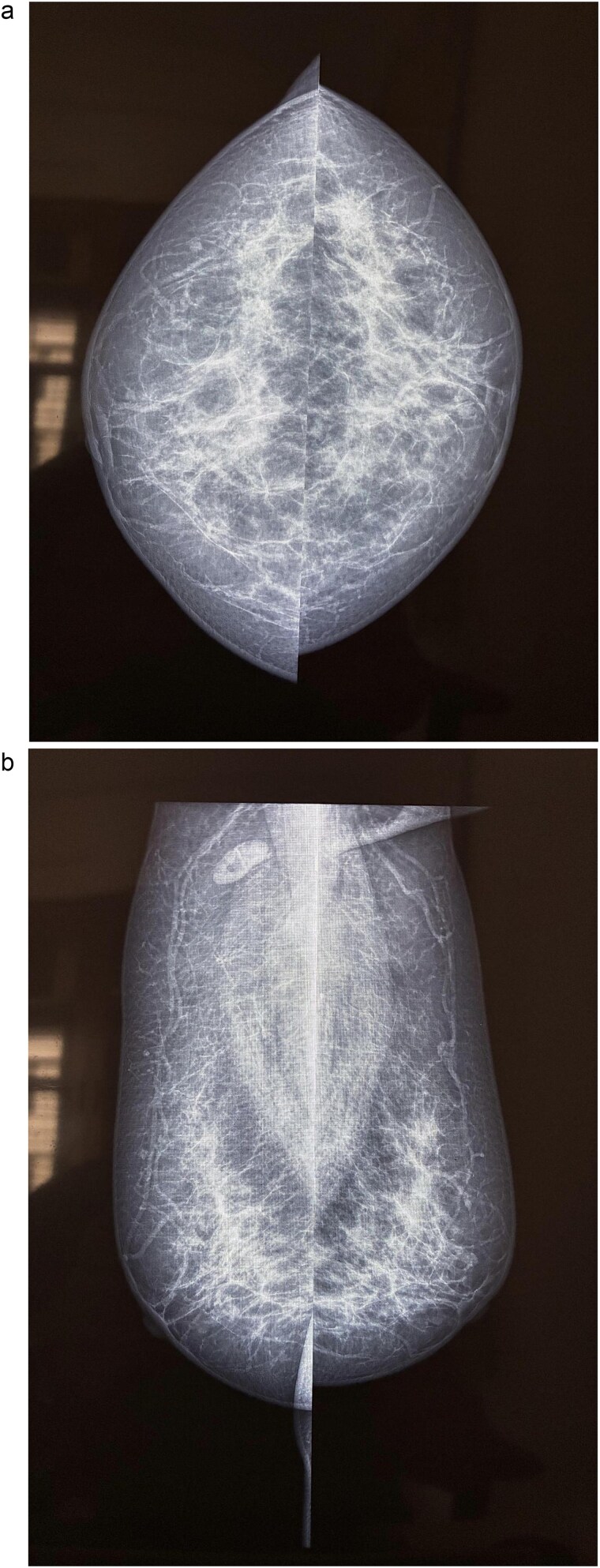
Mammography of the left breast. (a) Craniocaudal view showing a high density spiculated lesion in the upper outer quadrant, consistent with BIRADS 4c/5, suspicious for malignancy. (b) Mediolateral oblique view demonstrating the same spiculated, high density lesion in the upper outer quadrant, corresponding to the known carcinoma (BIRADS 4c/5).

**Figure 2:**
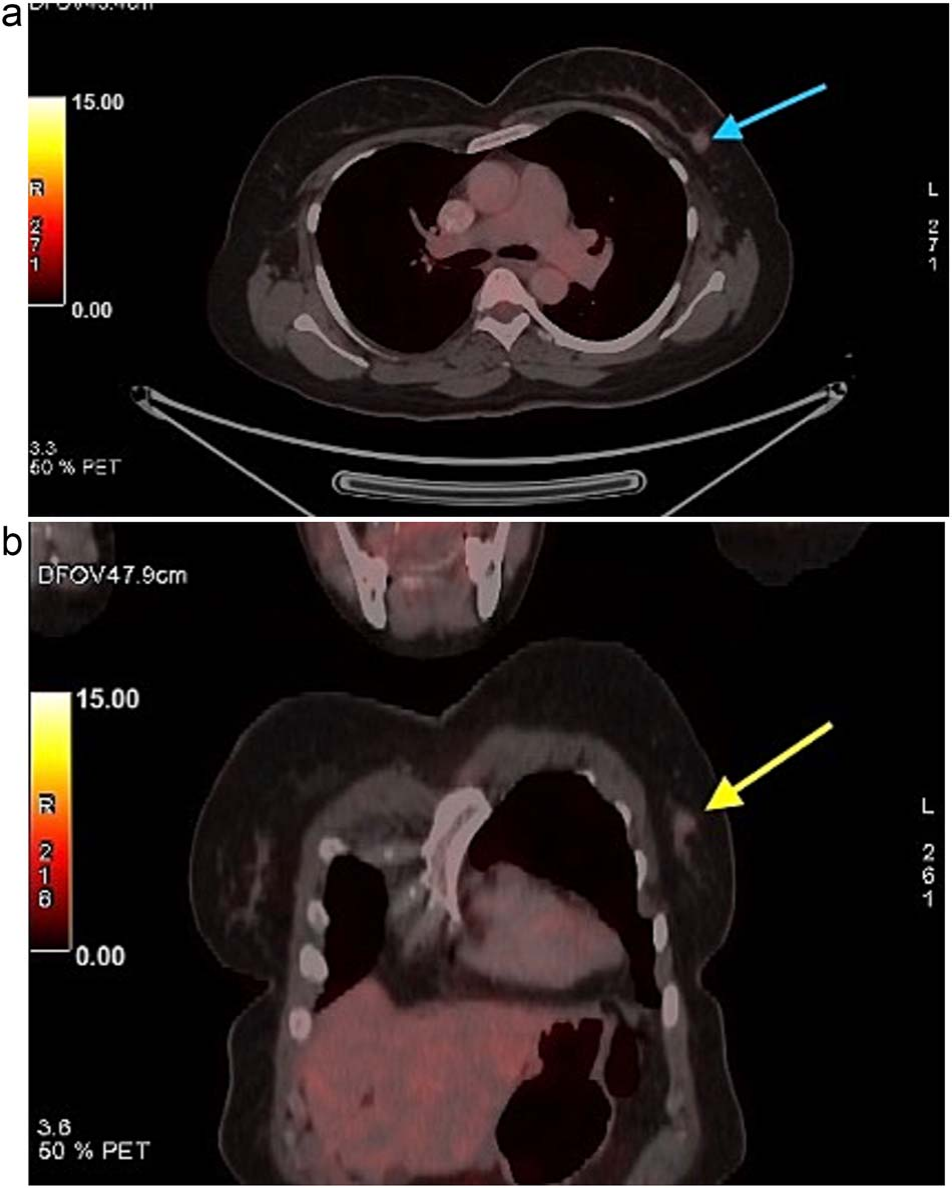
FDG PET CT images of the chest. (a) Axial PET CT image showing a well-defined FDG avid lesion in the upper outer quadrant of the left breast , consistent with the known carcinoma, with no evidence of axillary or mediastinal lymphadenopathy. (b) Coronal PET CT image demonstrating the left breast lesion in the upper outer quadrant without invasion into the chest wall or adjacent structures, consistent with localized disease.

A 58-year-old woman with no relevant family history underwent routine health screening during which a left breast lump was detected. Mammography showed a high density spiculated lesion in the upper outer quadrant (BIRADS 4c/5) (Fig. 1a and b). Ultrasound demonstrated a 1.0 × 1.1 cm spiculated, heterogeneous hypoechoic lesion (BIRADS 4c). FDG positron emission tomography computed tomography (PET CT) revealed mild uptake confined to the breast lesion (SUVmax ~2.5) with no FDG avid axillary nodes or distant disease (Fig. 2a and b). Ultrasound guided FNAC revealed infiltrating ductal carcinoma (Yokohama Category 5) with cohesive clusters and scattered malignant ductal epithelial cells showing nuclear overlapping, moderate pleomorphism, conspicuous nucleoli, and moderate cytoplasm in a hemorrhagic background.

**Figure 3:**
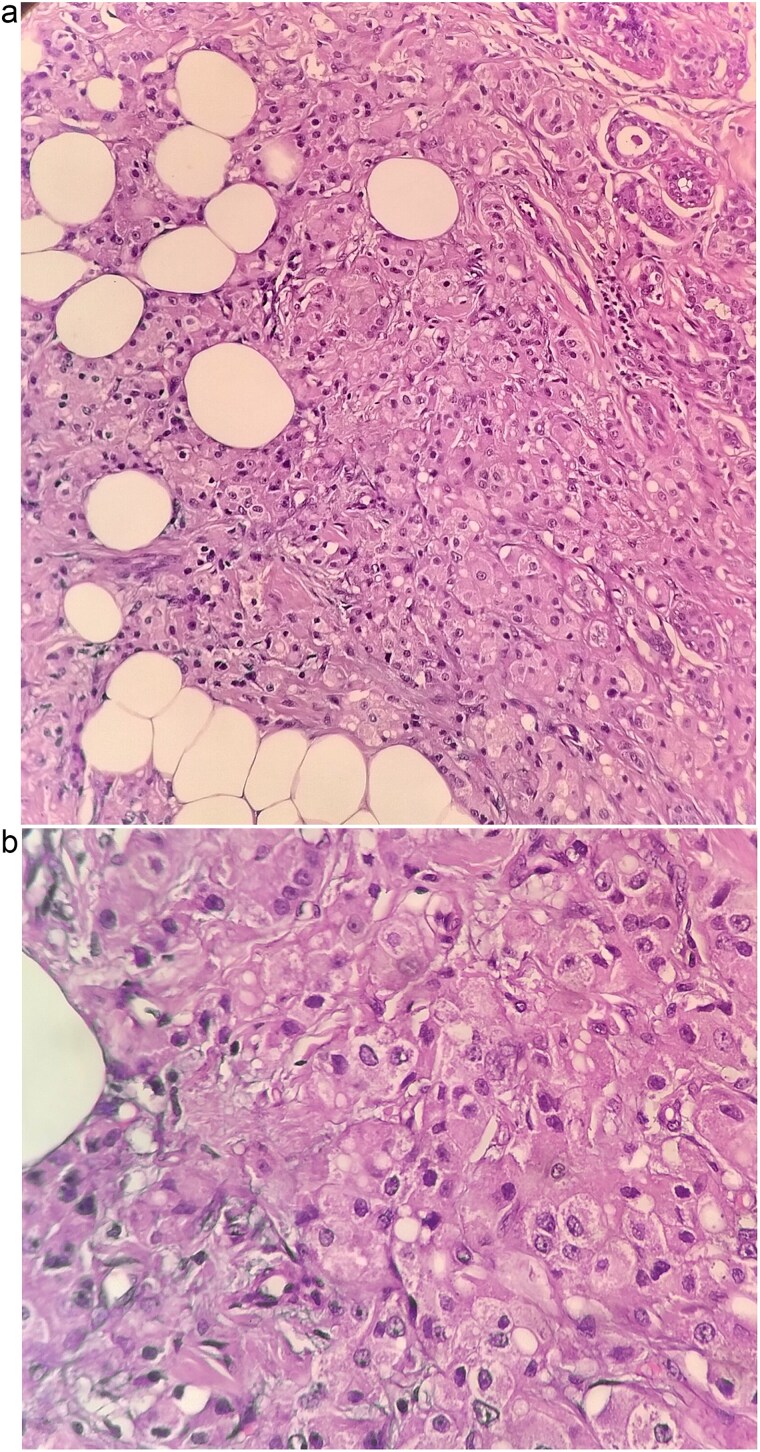
Histopathological sections of the breast lesion showing characteristic apocrine morphology. (a) H&E × 100: Tumor nests composed of large polygonal cells with abundant eosinophilic granular cytoplasm and prominent nucleoli infiltrating fibroadipose tissue. (b) H&E × 400: High power view highlighting the classic apocrine cytology with vesicular nuclei, prominent nucleoli, and abundant eosinophilic cytoplasm. These features are consistent with invasive apocrine carcinoma of the breast.

The patient underwent left breast conserving surgery with oncoplastic volume displacement and sentinel lymph node biopsy; five sentinel nodes were retrieved and all were negative intraoperatively and on final histology. Final histopathology demonstrated a 1.7 × 1 × 1 cm invasive carcinoma composed predominantly of large eosinophilic cells with vesicular nuclei, and focal low grade DCIS of cribriform and solid pattern (Fig. 3a and b). Margins were negative. The tumor was reported as carcinoma NST with apocrine differentiation, modified Bloom Richardson grade 2 (score 6). Immunohistochemistry showed ER, PR, and HER2 negativity, strong AR expression in 90% of tumor cells, Ki-67 8%, AE1/AE3, and CK7 positivity, TRPS1 focal positivity, and GATA3, DOG1 and SOX10 negativity.

Adjuvant treatment comprised whole breast radiotherapy of 40 Gy in 15 fractions over 3 weeks, followed by systemic chemotherapy with paclitaxel and carboplatin administered every 3 weeks for six cycles (paclitaxel 175 mg/m^2^ and carboplatin AUC 5, as tolerated). The choice of paclitaxel–carboplatin was made at the multidisciplinary tumor board as a balance between guideline based adjuvant chemotherapy for triple negative breast cancer and the relatively indolent behavior of low proliferative apocrine carcinoma. This regimen provided systemic coverage while avoiding an anthracycline in view of low Ki-67, small T1 size, and node negativity. She will be followed clinically every 3 months for the first 2 years, then every 6 months from year 2 to year 5, and annually thereafter. At 1-year follow up the patient remains disease free with no clinical or radiologic evidence of recurrence.

## Discussion

IAC represents a histologically and biologically distinct subtype of breast cancer. Unlike basal like triple negative breast cancers, these tumors often have lower Ki-67 proliferation indices, a more indolent course, and a strong association with androgen receptor expression [1–3]. Recognition of this distinction is essential for the surgical oncologist, as it influences operative planning, systemic therapy decisions, and follow up strategies.

A critical diagnostic challenge lies in the limited FDG uptake sometimes seen in low proliferative apocrine carcinomas. Reliance on PET avidity alone can therefore lead to underestimation of disease burden; histology and conventional breast imaging remain central to accurate staging [5]. Another frequent pitfall is misclassification as invasive ductal carcinoma NST when AR immunostaining is not performed in ER/PR negative cases. This misstep risks overlooking the apocrine phenotype and its therapeutic implications [3]. Cytological evaluation poses further difficulty, as apocrine cells are typically large with abundant eosinophilic granular cytoplasm and prominent nucleoli, while usual ductal carcinoma cells are smaller [6]. Without awareness of these features and correlation with IHC, misclassification is possible.

Surgical management remains the cornerstone for localized IAC. Breast conserving surgery is appropriate when margins are clear, and oncoplastic techniques permit wider excisions while maintaining cosmesis. Particular attention should be paid to the coexistence of apocrine DCIS, where cavity shave margins and careful orientation may help reduce re-excision rates. Sentinel lymph node biopsy is adequate for staging in clinically node negative patients, and surgeons should avoid routine axillary dissection when sentinel nodes are negative or when fewer than three nodes are positive. This approach prevents overtreatment while remaining oncologically safe, consistent with modern evidence supporting conservative axillary surgery [7].

Systemic therapy for IAC is an evolving area. Although chemotherapy is generally recommended for triple negative breast cancers, the decision should be individualized for apocrine tumors that demonstrate low proliferation (low Ki-67) and favorable clinicopathologic features. In our multidisciplinary tumor board, given the tumor’s T1 size, node negativity, and low Ki-67 (8%), we selected paclitaxel-carboplatin as adjuvant therapy to align with TNBC systemic coverage while avoiding anthracycline exposure. Platinum containing regimens are supported in certain TNBC subsets and in BRCA associated disease [8]. When Ki-67 is high or adverse features (larger size, nodal involvement) are present, escalation to include anthracycline containing regimens is considered in line with standard TNBC practice [9].

AR directed therapy (e.g. enzalutamide, bicalutamide) has shown clinical activity in AR positive TNBC, particularly in the metastatic setting [9–11]. There is currently no established role for routine adjuvant AR blockade in the curative setting; such agents are reserved for advanced disease or clinical trial contexts.

Similarly, PI3K/AKT/mTOR pathway alterations (e.g. PIK3CA or AKT mutations) provide rationale for combining AR blockade with targeted inhibitors, although these strategies remain investigational [10, 11].

Genetic testing has a role in management planning. Current guideline recommendations for germline BRCA1/2 testing are based on tumor phenotype and family history rather than age alone. For triple negative disease, testing is indicated regardless of age, especially if other risk factors are present. Identifying a pathogenic germline variant would have systemic implications, particularly for PARP inhibitor use, as well as preventive relevance for family members.

Surveillance following treatment of IAC should follow standard breast oncology protocols. FDG PET CT is not reliable for recurrence detection in low proliferative apocrine tumors, reinforcing the importance of structured clinical follow up and mammographic surveillance.

In summary, IAC requires nuanced surgical and systemic decision-making. The surgeon’s role extends beyond resection to ensuring accurate diagnosis, avoiding unnecessary axillary procedures, and advocating for AR testing to individualize therapy. Precision in these aspects not only prevents overtreatment but also positions patients for future targeted and trial based therapies.

## References

[1] Farmer P, Bonnefoi H, Anderle P, et al. Identification of molecular apocrine breast tumours by microarray analysis. Oncogene 2005;24:4660–71. 15897907 10.1038/sj.onc.1208561

[2] WHO Classification of Tumours Editorial Board . Breast Tumours, 5th edn. Lyon: International Agency for Research on Cancer (IARC), 2019.

[3] Niemeier LA, Dabbs DJ, Beriwal S, et al. Androgen receptor in breast cancer: expression in ER-positive tumors and apocrine carcinomas. Mod Pathol 2010;23:205–12. 19898421 10.1038/modpathol.2009.159

[4] Gucalp A, Tolaney S, Isakoff SJ, et al. Phase II trial of bicalutamide in androgen receptor–positive, ER/PgR-negative metastatic breast cancer. Clin Cancer Res 2013;19:5505–12. 23965901 10.1158/1078-0432.CCR-12-3327 PMC4086643

[5] Koolen BB, Valdés Olmos RA, Elkhuizen PH, et al. Locoregional staging of breast cancer patients with (18F)FDG PET/CT: added value and pitfalls. Breast Cancer Res Treat 2012;131:117–26.21935602

[6] Tolkach Y, Wöckel A, Mittelbronn M, et al. Apocrine carcinoma of the breast: a review of 42 cases, including cytological and histological characteristics. Mod Pathol 2014;27:1697–705.

[7] Giuliano AE, Ballman KV, McCall L, et al. Effect of axillary dissection vs sentinel node biopsy on survival in breast cancer with sentinel node metastasis: the ACOSOG Z0011 trial. JAMA. 2011;305:569–75. 21304082 10.1001/jama.2011.90 PMC5389857

[8] Tutt A, Garber JE, Kaufman B, et al. Carboplatin in BRCA1/2-mutated and triple-negative breast cancer: the TNT trial. Lancet Oncol 2018;19:475–89.

[9] Traina TA, Miller K, Yardley DA, et al. Enzalutamide for the treatment of androgen receptor–expressing triple-negative breast cancer. J Clin Oncol 2018;36:884–90. 29373071 10.1200/JCO.2016.71.3495 PMC5858523

[10] Lehmann BD, Bauer JA, Schafer JM, et al. PIK3CA mutations in androgen receptor–positive triple-negative breast cancer confer sensitivity to enzalutamide. Breast Cancer Res 2014;16:R65. 25103565 10.1186/s13058-014-0406-x PMC4187324

[11] Chia K, O’Brien M, Brown M, et al. Targeting the androgen receptor in breast cancer. Curr Oncol Rep 2015;17:4. 25665553 10.1007/s11912-014-0427-8

